# Crystal structure of [bis­(2-amino­ethyl-κ*N*)(2-{[4-(tri­fluoro­meth­yl)benzyl­idene]amino}­eth­yl)amine-κ*N*]di­chlorido­copper(II)

**DOI:** 10.1107/S2056989015024147

**Published:** 2016-01-01

**Authors:** Katherine A. Bussey, Annie R. Cavalier, Margaret E. Mraz, Ashley S. Holderread, Kayode D. Oshin, Allen G. Oliver, Matthias Zeller

**Affiliations:** a Department of Chemistry & Physics, Saint Marys College, Notre Dame, IN 46556, USA; b Department of Chemistry & Biochemistry, University of Notre Dame, Notre Dame, IN 46556, USA; c Department of Chemistry, Youngstown State University, Youngstown, OH 44555, USA

**Keywords:** crystal structure, four-coordinate copper(II) complex, tri­fluoro­methyl group analogue, ligand disorder

## Abstract

The Cu^II^ atom in the title compound, [CuCl_2_(C_14_H_21_F_3_N_4_)], adopts a coordination geometry that is between distorted square-based pyramidal and very Jahn–Teller-elongated octa­hedral. It is coordinated by three N atoms from the bis­(2-amino­eth­yl)(2-{[4-(tri­fluoro­meth­yl)benzyl­idene]amino}­eth­yl)amine and two chloride ligands. The two crystallographically unique copper complexes present in the asymmetric unit exhibit noticeable differences in the coordination bond lengths. Considering the Cu^II^ atoms as having square-pyramidal geometry, the basal Cu—Cl bond lengths are typical [2.2701 (12) and 2.2777 (12) Å], while the apical distances are considerably elongated [2.8505 (12) and 2.9415 (12) Å]. For each mol­ecule, a Cu^II^ atom from inversion-related mol­ecules are in nearby proximity to the remaining axial Cu^II^ sites, but the Cu⋯Cl distances are very long [3.4056 (12) and 3.1645 (12) Å], attributable to van der Waals contacts. Nonetheless, these contacts appear to have some structure-directing properties, leading to association into dimers. These dimers associate *via* stacking of the aromatic rings to form extended zigzag chains.

## Abstract 2

The asymmetric unit of the title compound contains two crystallographically unique copper complexes. In each complex, the Cu atom is bound to two chloride ligands and to three N atoms of the 2-(4-tri­fluoro­methyl­benzyl­idene­amino)­ethyl­amine-bis­(2–2amino­eth­yl)amine ligand to give a distorted square-based pyramidal geometry in which the axial Cu—Cl bond is elongated, indicative of Jahn–Teller distortion. A Cu atom from a symmetry-related mol­ecule is in nearby proximity to the remaining axial Cu site, thus the overall geometry about each Cu atom could be described as being between distorted square-based pyramidal and very elongated Jahn–Teller-distorted octa­hedral.

## Chemical context   

The introduction of a fluorine atom or perfluoro­alkyl group into a compound can bring about significant changes in its physical, chemical, and biological properties, making organo-fluorine derivatives suitable for diverse applications in areas of material science, agrochemistry, and medicinal chemistry (Singh & Shreeve, 2000 ▸). Modifications include polarity and conformational changes, increased chemical or metabolic stability, and enhanced lipophilicity (Böhm *et al.*, 2004 ▸). As many as 30–40% of agrochemicals and 20% of pharmaceuticals on the market are estimated to contain fluorine, including three of the top eight drugs sold in 2007 (Dubinia *et al.*, 2008 ▸). Fluorination can also serve as a diagnostic tool, enabling techniques such as ^19^F NMR spectroscopy and positron emission tomography, with some organo-fluorine compounds exhibiting inter­esting NMR spectra (Purser *et al.*, 2008 ▸). The simplest perfluoro­alkyl group, tri­fluoro­methyl, has become an important structural component for many compounds, mainly because of its polar influence and effect on lipophilicity (Dolbier, 2009 ▸). Its electronegativity and relatively small size (only two and one-half times the volume of a methyl group) contribute to this behavior (Welch, 1987 ▸). As such, synthesis of simple and complex compounds incorporating fluorinated analogues of the methyl group has become a growing area of inter­est. In this context, we report the synthesis and crystal structure of the title compound [CuCl_2_(C_14_H_21_N_4_F_3_)] (**1**).
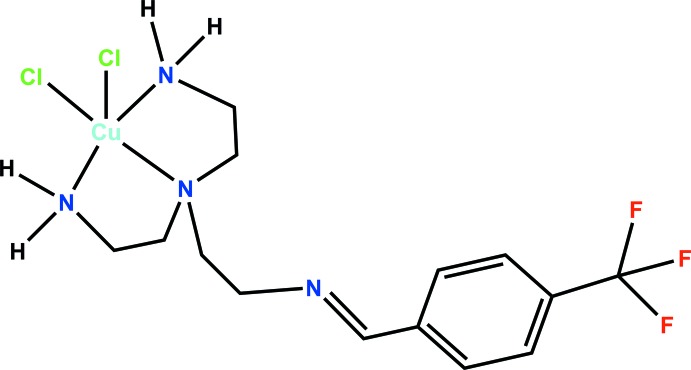


## Structural commentary   

**Figure 1:**
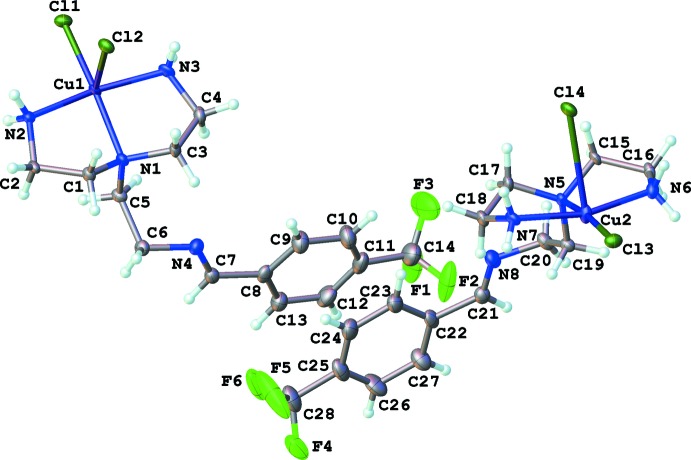
Asymmetric unit of the title compound, showing atomic displacement ellipsoids at the 50% probability level and the atom-numbering scheme.

**Figure 2:**
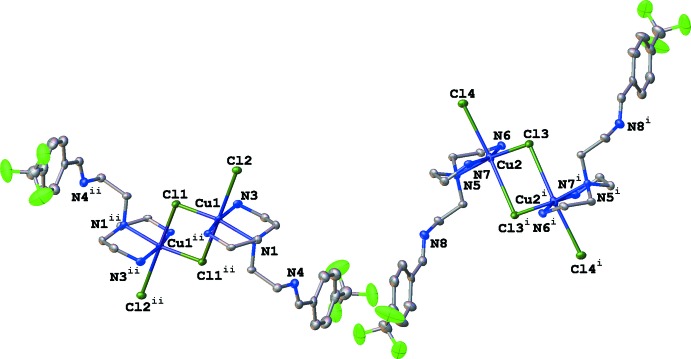
Dimer inter­actions between [CuCl_2_(C_14_H_21_N_4_F_3_)] mol­ecules, shown with 50% probability ellipsoids. H atoms were removed for clarity. Symmetry codes: (i) −*x* + 1, −*y* + 1, −*z*; (ii) −*x*, −*y*, −*z* + 2.

The asymmetric unit of the title compound contains two Cu–ligand complexes (Fig. 1 ▸). The coordination geometries of both Cu^II^ ions are between distorted square-based pyramidal and very Jahn–Teller-distorted octa­hedral. The first complex displays Cu—Cl bond lengths of 2.2701 (12) and 2.8505 (12) Å, while Cu—Cl lengths of 2.2777 (12) and 2.9415 (12) Å are observed in the second (Table 1 ▸, Fig. 2 ▸). Some studies suggest that copper(II) complexes adopting square-pyramidal geometries with apical Cu—*L* bonds longer than the basal bonds by up to 0.5 Å may not be due to Jahn–Teller distortion, but the result of a double electron occupancy of the anti­bonding *a_1_* orbital and single occupancy of the *b_1_* orbital, leading to increased anti-bonding electron density along the apical Cu—*L* axis (Rossi & Hoffmann, 1975 ▸). Copper(II) complexes with a square plane of ligand donors and one or two axial Cu—*L* inter­actions of 2.1–2.8 Å are very common (Murphy & Hathaway, 2003 ▸). Taking into consideration the covalent and van der Waals radii of copper (1.4 Å), an axial Cu—Cl bond length of less than 2.8 Å can be viewed as a genuine bond while bond lengths between 2.8–3.2 Å represent a weaker secondary inter­action that is predominantly electrostatic in nature. Distances greater than 3.2 Å can be considered as purely van der Waals contacts (Halcrow, 2013 ▸). Following these criteria, it would seem that the inter­action observed between Cu2⋯Cl3^ii^ [3.1645 (12) Å; symmetry code: (ii) −*x*, −*y*, −*z* + 2] is a weaker secondary inter­action with electrostatic characteristics. However, an elongated Cu1⋯Cl2^i^ distance of 3.4056 (12) Å is also observed, which can be attributed to a van der Waals contact [Halcrow, 2013 ▸; symmetry code: (i) −*x* + 1, −*y* + 1, −*z*). These contacts appear to have some structure-directing properties, producing chlorine-bridged dimers in the crystal structure of (**1**).

## Supra­molecular features   

**Figure 3:**
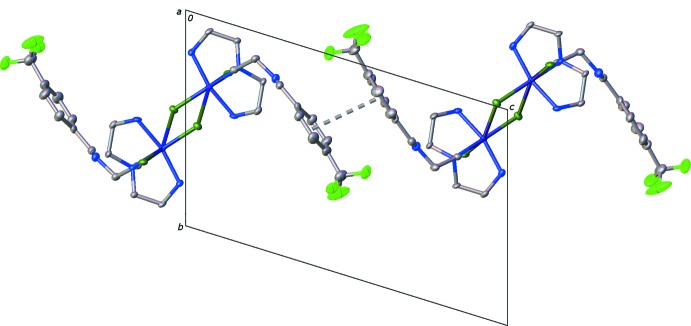
View along the *a* axis showing weak inter­molecular inter­actions present in the crystal lattice. Atomic displacement ellipsoids depicted at 50% probability level with π–π inter­actions shown as dashed gray lines.

In addition to electrostatic inter­actions observed in each complex, the aromatic rings engage in offset face-to-face π–π inter­actions with an observed centroid-to-centroid distance of 3.906 (3) Å and a dihedral angle of 10.6 (3)° (Fig. 3 ▸). Inspection of the extended structure shows that the orientation of these phenyl rings (C8–C13 and C22–C27) reduces inter­actions of the CF_3_ groups associated with these rings. Coupled with the chlorine-bridged dimer we find that chains of mol­ecules extend through the crystal parallel to the [221] direction (Fig. 3 ▸).

Inspection of inter­molecular/intra­molecular contacts reveals that amine nitro­gen atoms N2, N3, N6 and N7 are involved in N—H⋯Cl hydrogen-bonds (Table 2 ▸). However, several of the contacts [N3⋯Cl3^i^, N3⋯Cl2, N7⋯Cl4; symmetry code: (i) −*x* + 1, −*y* + 1, −*z*] have severely constrained N—H⋯Cl angles and are merely contacts to chlorine atoms bonded to the same Cu^II^ atom. The remaining hydrogen-bond contacts are inter­molecular inter­actions, and while relatively long, they likely contribute to structure-directed organization.

## Database survey   

There are 318 structures that incorporate the *N*-2-bis(2-amino­eth­yl)amino­ethyl ligand skeleton (Groom & Allen, 2014 ▸; CSD Version 5.36). Of those 318 structures, five incorporate one *para*-substituted benzene ring as presented in this article. Of those five, two have bromo-substituted phenyl rings displaying a nickel metal atom with perchlorate counter-ion and a zinc metal atom with tetra­fluorido­borate counter-ion, respectively. Two display nitro-substituted phenyl rings with a copper metal atom and perchlorate counter-ions. Of those two, one contains a bidentate ligand with an ammonium derivative group not coordinating to the metal atom. The final structure is a zinc complex incorporating an unsubstituted phenyl ring with a perchlorate counter-ion. Of the 318 structures, none incorporates the tri­fluoro­methyl-substituted phenyl group presented here and none displays the dichloride counterions presented here. A survey of Cu—Cl bond-length elongations of similar structures in the literature produced examples such as 2.6061 (18) and 2.609 (2) Å (Tucker *et al.*, 1991 ▸), 2.843 (1) to 3.140 (1) Å (Krysiak *et al.*, 2014 ▸), 2.665 (3) and 2.731 (2) Å (Ferrari *et al.*, 1998 ▸) and 2.7546 (9) Å (Odoko *et al.*, 2002 ▸).

## Synthesis and crystallization   

**Figure 4:**
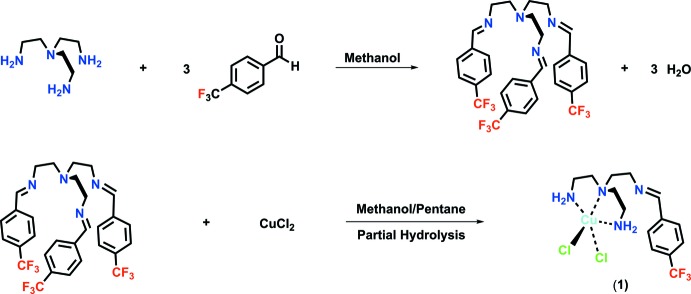
Synthetic scheme for [Cu(C_14_H_21_N_4_Cl_2_F_3_)(Cl_2_)]

**Synthesis of** **tris(2-(4-tri­fluoro­methyl­benzyl­idene­amino)­eth­yl)amine ligand:** In a drybox, *tris*(2-(amino)­eth­yl)amine (2.56 mL, 17.10 mmol) was dissolved in 100 mL methanol in a 250 mL round-bottom flask (Fig. 4 ▸). Ligand precursor 4-(tri­fluoro­meth­yl)benzaldehyde (6.90 mL, 51.29 mmol) was added to the flask to give a light-yellow colored solution. Reaction was sealed and allowed to mix for 48 h producing a clear yellow solution. Solvent was removed using a rotary evaporator and dried under vacuum for one h to yield a yellow solid (10.40 g, 99%). ^1^H NMR (CDCl_3_, 500 MHz): δ 2.94 (*t*, *J* = 7.6 Hz, 2H), 3.70 (*t*, *J* = 7.5 Hz, 2H), 7.56 (*br*, 2H), 8.08 (*s*, 1H). ^13^C NMR (CDCl_3_, 500 MHz): δ 55.62, 60.32, 122.85 (*q*), 125.73 (*q*), 128.35, 132.44 (*q*), 139.62, 160.42. FT–IR (solid) *v* (cm^−1^): 1321 (*s*), 1169 (*s*), 1118 (*s*), 1062 (*s*), 834 (*s*). Melting Point: 344 K. TOF–ESI–MS: (*m*/*z*) [*M* + (H)]^+^ calculated for C_30_H_28_N_4_F_9_ = 615.2165, found 615.2194 (4.8 p.p.m.).

**Synthesis of 2-(4-tri­fluoro­methyl­benzyl­idene­amino)­eth­yl)amine-bis(2-αminoeth­yl)amine copper(II) chloride complex:** *tris*(2-(4-Tri­fluoro­methyl­benzyl­idene­amino)­eth­yl)amine (1.000 g, 1.63 mmol) was dissolved in 20 mL methanol in a 100 mL round-bottom flask. CuCl_2_ (0.219 g, 1.63 mmol) was added to the flask to give a teal-colored solution. The reaction was allowed to mix for six h then 20 mL of pentane was slowly added to the solution to generate a teal-colored precipitate. Solvent was removed from the round-bottom flask by connecting it to a rotary evaporator. The precipitate obtained was washed twice by transferring 15 mL of pentane into the flask and stirring vigorously for thirty minutes. Solvent was removed and precipitate dried under vacuum for one h to yield a teal-colored solid (1.140 g, 93%). FT–IR (solid): *v* (cm^−1^) = 1636 (*m*), 1506 (*s*), 1473 (*s*), 1317 (*s*), 1163 (*s*), 1109 (*br*), 830 (*s*). UV–Vis (MeOH) λ_max_ = 668 nm. TOF–ESI–MS: (*m*/*z*) [*M* – 2(Cl)]^2+^ calculated for C_30_H_27_N_4_F_9_Cu = 677.1383, found 677.1381 (0.2 p.p.m.). Blue single crystal plates suitable for X-ray analysis were obtained by slow diffusion of diethyl ether into a complex solution made in aceto­nitrile at room temperature. The structure obtained is indicative of hydrolysis occuring on two amine positions of the intended copper(II) complex.

## Refinement   

Crystal data, data collection and structure refinement details are summarized in Table 3 ▸. Hydrogen atoms were placed at calculated positions and constrained to ride on their parent atoms with *U*_iso_(H) = 1.2*U*_eq_(C,N) for methyl­ene, aromatic and amide groups with C—H distances set at 0.99 Å (methyl­ene), 0.95 Å (aromatic) and N—H = 0.91 Å.

## Supplementary Material

Crystal structure: contains datablock(s) global, I. DOI: 10.1107/S2056989015024147/pk2570sup1.cif

Structure factors: contains datablock(s) I. DOI: 10.1107/S2056989015024147/pk2570Isup2.hkl

Supporting information file. DOI: 10.1107/S2056989015024147/pk2570Isup3.pdf

CCDC reference: 1442779

Additional supporting information:  crystallographic information; 3D view; checkCIF report

## Figures and Tables

**Table 1:** Selected geometric parameters (Å, °)

Cu1—N2	1.986 (3)	Cu2—N7	1.986 (4)
Cu1—N3	1.988 (4)	Cu2—N6	1.989 (4)
Cu1—N1	2.062 (4)	Cu2—N5	2.070 (4)
Cu1—Cl1	2.2701 (12)	Cu2—Cl3	2.2777 (12)
Cu1—Cl2	2.8505 (12)	Cu2—Cl4	2.9415 (12)
Cu1—Cl1^i^	3.4056 (12)	Cu2—Cl3^ii^	3.1645 (12)
			
N2—Cu1—N3	166.47 (15)	N7—Cu2—N6	163.80 (16)
N2—Cu1—N1	84.81 (14)	N7—Cu2—N5	85.50 (15)
N3—Cu1—N1	85.31 (14)	N6—Cu2—N5	85.18 (14)
N2—Cu1—Cl1	95.85 (11)	N7—Cu2—Cl3	95.55 (11)
N3—Cu1—Cl1	95.68 (11)	N6—Cu2—Cl3	95.56 (11)
N1—Cu1—Cl1	168.47 (11)	N5—Cu2—Cl3	171.82 (11)
N2—Cu1—Cl2	88.27 (11)	N7—Cu2—Cl4	81.07 (11)
N3—Cu1—Cl2	83.37 (11)	N6—Cu2—Cl4	86.35 (11)
N1—Cu1—Cl2	94.77 (10)	N5—Cu2—Cl4	93.74 (10)
Cl1—Cu1—Cl2	96.76 (4)	Cl3—Cu2—Cl4	94.44 (4)
N2—Cu1—Cl1^i^	115.18 (11)	N7—Cu2—Cl3^ii^	80.52 (11)
N3—Cu1—Cl1^i^	74.19 (11)	N6—Cu2—Cl3^ii^	113.16 (12)
N1—Cu1—Cl1^i^	90.95 (10)	N5—Cu2—Cl3^ii^	92.88 (10)
Cl1—Cu1—Cl1^i^	78.32 (4)	Cl3—Cu2—Cl3^ii^	79.32 (4)
Cl2—Cu1—Cl1^i^	156.30 (3)	Cl4—Cu2—Cl3^ii^	159.87 (3)

Symmetry codes: (i) 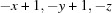; (ii) 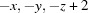.

**Table 2:** Hydrogen-bond geometry (Å, °)

*D*—H⋯*A*	*D*—H	H⋯*A*	*D*⋯*A*	*D*—H⋯*A*
N2—H2*A*⋯Cl3^iii^	0.91	2.84	3.591 (4)	141
N2—H2*B*⋯Cl4^iv^	0.91	2.46	3.365 (4)	171
N3—H3*A*⋯Cl1^i^	0.91	2.95	3.444 (4)	115
N3—H3*A*⋯Cl4^v^	0.91	2.60	3.334 (4)	139
N3—H3*B*⋯Cl2	0.91	2.83	3.281 (4)	112
N6—H6*C*⋯Cl1^v^	0.91	2.96	3.681 (4)	138
N6—H6*D*⋯Cl2^vi^	0.91	2.45	3.342 (4)	167
N7—H7*A*⋯Cl2^iii^	0.91	2.57	3.348 (4)	143
N7—H7*B*⋯Cl4	0.91	2.79	3.284 (4)	115

Symmetry codes: (i) 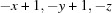; (iii) 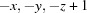; (iv) 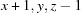; (v) 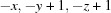; (vi) 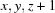.

**Table 3:** Experimental details

Crystal data	
Chemical formula	[CuCl_2_(C_14_H_21_F_3_N_4_)]
*M*_r_	436.80
Crystal system, space group	Triclinic, *P* 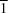
Temperature (K)	100
*a*, *b*, *c* (Å)	9.8506 (6), 11.0603 (7), 17.8574 (12)
α, β, γ (°)	73.110 (3), 75.530 (2), 89.010 (2)
*V* (Å^3^)	1799.4 (2)
*Z*	4
Radiation type	Mo *K*α
μ (mm^−1^)	1.54
Crystal size (mm)	0.30 × 0.19 × 0.05

Data collection	
Diffractometer	Bruker AXS D8 Quest CMOS
Absorption correction	Multi-scan (*SADABS*; Bruker, 2013 ▸)
*T*_min_, *T*_max_	0.573, 0.746
No. of measured, independent and observed [*I* > 2σ(*I*)] reflections	49937, 8937, 7063
*R*_int_	0.079

Refinement	
*R*[*F* ^2^ > 2σ(*F* ^2^)], *wR*(*F* ^2^), *S*	0.073, 0.151, 1.22
No. of reflections	8937
No. of parameters	433
H-atom treatment	H-atom parameters constrained
Δρ_max_, Δρ_min_ (e Å^−3^)	1.32, −0.74

Computer programs: *APEX2* and *SAINT* (Bruker, 2013 ▸), *SHELXS97* (Sheldrick, 2008 ▸), *SHELXL* (Sheldrick, 2015 ▸), *CrystalMaker* (Palmer, 2007 ▸) and *OLEX2* (Dolomanov *et al.*, 2009 ▸), *publCIF* (Westrip, 2010 ▸), and *PLATON* (Spek, 2009 ▸).

## supplementary crystallographic information

### Crystal data

**Table:**

[CuCl_2_(C_14_H_21_F_3_N_4_)]	*Z* = 4
*M**_r_* = 436.80	*F*(000) = 892
Triclinic, *P*1	*D*_x_ = 1.612 Mg m^−^^3^
*a* = 9.8506 (6) Å	Mo *K*α radiation, λ = 0.71073 Å
*b* = 11.0603 (7) Å	Cell parameters from 9961 reflections
*c* = 17.8574 (12) Å	θ = 2.5–28.3°
α = 73.110 (3)°	µ = 1.54 mm^−^^1^
β = 75.530 (2)°	*T* = 100 K
γ = 89.010 (2)°	Plate, blue
*V* = 1799.4 (2) Å^3^	0.30 × 0.19 × 0.05 mm

### Data collection

**Table:**

Bruker AXS D8 Quest CMOS diffractometer	8937 independent reflections
Radiation source: I-mu-S microsource X-ray tube	7063 reflections with *I* > 2σ(*I*)
Laterally graded multilayer (Goebel) mirror monochromator	*R*_int_ = 0.079
ω and phi scans	θ_max_ = 28.3°, θ_min_ = 2.5°
Absorption correction: multi-scan (*SADABS*; Bruker, 2013)	*h* = −13→13
*T*_min_ = 0.573, *T*_max_ = 0.746	*k* = −14→14
49937 measured reflections	*l* = −23→23

### Refinement

**Table:**

Refinement on *F*^2^	Primary atom site location: structure-invariant direct methods
Least-squares matrix: full	Secondary atom site location: difference Fourier map
*R*[*F*^2^ > 2σ(*F*^2^)] = 0.073	Hydrogen site location: inferred from neighbouring sites
*wR*(*F*^2^) = 0.151	H-atom parameters constrained
*S* = 1.22	*w* = 1/[σ^2^(*F*_o_^2^) + (0.0341*P*)^2^ + 8.3032*P*] where *P* = (*F*_o_^2^ + 2*F*_c_^2^)/3
8937 reflections	(Δ/σ)_max_ = 0.001
433 parameters	Δρ_max_ = 1.32 e Å^−^^3^
0 restraints	Δρ_min_ = −0.74 e Å^−^^3^

### Special details

**Table:**

Geometry. All e.s.d.'s (except the e.s.d. in the dihedral angle between two l.s. planes) are estimated using the full covariance matrix. The cell e.s.d.'s are taken into account individually in the estimation of e.s.d.'s in distances, angles and torsion angles; correlations between e.s.d.'s in cell parameters are only used when they are defined by crystal symmetry. An approximate (isotropic) treatment of cell e.s.d.'s is used for estimating e.s.d.'s involving l.s. planes.

### Fractional atomic coordinates and isotropic or equivalent isotropic displacement parameters (Å^2^)

**Table:**

	*x*	*y*	*z*	*U*_iso_*/*U*_eq_	
Cu1	0.37014 (6)	0.32211 (5)	0.07300 (3)	0.01576 (13)	
Cl1	0.34574 (11)	0.47852 (10)	−0.03695 (7)	0.0181 (2)	
Cl2	0.08223 (11)	0.23298 (9)	0.13230 (7)	0.0178 (2)	
N1	0.4341 (4)	0.1932 (3)	0.1650 (2)	0.0148 (7)	
N2	0.4034 (4)	0.1876 (3)	0.0182 (2)	0.0153 (7)	
H2A	0.3259	0.1748	0.0018	0.018*	
H2B	0.4768	0.2135	−0.0265	0.018*	
N3	0.3162 (4)	0.4228 (3)	0.1506 (2)	0.0180 (8)	
H3A	0.3559	0.5029	0.1269	0.022*	
H3B	0.2213	0.4275	0.1638	0.022*	
N4	0.6465 (4)	0.1886 (4)	0.2797 (2)	0.0220 (8)	
C1	0.3888 (5)	0.0696 (4)	0.1598 (3)	0.0165 (8)	
H1A	0.2853	0.0565	0.1796	0.020*	
H1B	0.4309	0.0006	0.1945	0.020*	
C2	0.4347 (4)	0.0663 (4)	0.0728 (3)	0.0157 (8)	
H2C	0.5367	0.0540	0.0581	0.019*	
H2D	0.3841	−0.0054	0.0666	0.019*	
C3	0.3557 (5)	0.2219 (4)	0.2404 (3)	0.0173 (9)	
H3C	0.3981	0.1805	0.2855	0.021*	
H3D	0.2566	0.1893	0.2555	0.021*	
C4	0.3632 (5)	0.3641 (4)	0.2247 (3)	0.0200 (9)	
H4A	0.3018	0.3866	0.2712	0.024*	
H4B	0.4607	0.3951	0.2175	0.024*	
C5	0.5900 (4)	0.2083 (4)	0.1504 (3)	0.0171 (9)	
H5A	0.6154	0.2982	0.1425	0.020*	
H5B	0.6343	0.1892	0.0993	0.020*	
C6	0.6539 (5)	0.1267 (4)	0.2166 (3)	0.0192 (9)	
H6A	0.6021	0.0428	0.2407	0.023*	
H6B	0.7530	0.1136	0.1927	0.023*	
C7	0.7616 (5)	0.2140 (4)	0.2922 (3)	0.0207 (9)	
H7	0.8456	0.1851	0.2650	0.025*	
C8	0.7687 (5)	0.2881 (4)	0.3484 (3)	0.0229 (10)	
C9	0.6476 (6)	0.3277 (5)	0.3926 (3)	0.0317 (12)	
H9	0.5578	0.2990	0.3917	0.038*	
C10	0.6578 (6)	0.4083 (6)	0.4375 (4)	0.0380 (13)	
H10	0.5750	0.4342	0.4679	0.046*	
C11	0.7877 (7)	0.4513 (5)	0.4381 (3)	0.0339 (12)	
C12	0.9094 (6)	0.4099 (6)	0.3972 (4)	0.0365 (13)	
H12	0.9987	0.4374	0.3993	0.044*	
C13	0.8989 (6)	0.3276 (5)	0.3529 (3)	0.0296 (11)	
H13	0.9818	0.2978	0.3254	0.036*	
C14	0.7966 (7)	0.5463 (6)	0.4827 (4)	0.0439 (15)	
F1	0.9161 (5)	0.6165 (4)	0.4552 (2)	0.0643 (13)	
F2	0.7837 (6)	0.4938 (4)	0.5606 (2)	0.0708 (14)	
F3	0.6983 (6)	0.6312 (5)	0.4752 (4)	0.093 (2)	
Cu2	−0.05556 (6)	0.17013 (5)	0.92597 (3)	0.01768 (14)	
Cl3	−0.18291 (11)	0.01387 (10)	1.03343 (7)	0.0185 (2)	
Cl4	−0.30093 (11)	0.26904 (10)	0.86714 (7)	0.0194 (2)	
N5	0.0850 (4)	0.3019 (3)	0.8338 (2)	0.0139 (7)	
N6	−0.0796 (4)	0.3026 (4)	0.9827 (2)	0.0175 (7)	
H6C	−0.1725	0.3161	0.9980	0.021*	
H6D	−0.0480	0.2753	1.0281	0.021*	
N7	−0.0460 (4)	0.0769 (3)	0.8452 (2)	0.0172 (8)	
H7A	−0.0292	−0.0054	0.8669	0.021*	
H7B	−0.1297	0.0788	0.8320	0.021*	
N8	0.4024 (4)	0.3011 (4)	0.7164 (2)	0.0242 (9)	
C15	0.0291 (5)	0.4249 (4)	0.8417 (3)	0.0162 (8)	
H15A	−0.0582	0.4383	0.8232	0.019*	
H15B	0.0986	0.4951	0.8073	0.019*	
C16	−0.0011 (5)	0.4240 (4)	0.9293 (3)	0.0170 (9)	
H16A	0.0882	0.4326	0.9437	0.020*	
H16B	−0.0576	0.4963	0.9372	0.020*	
C17	0.0714 (5)	0.2776 (4)	0.7581 (3)	0.0176 (9)	
H17A	0.1522	0.3193	0.7125	0.021*	
H17B	−0.0158	0.3123	0.7447	0.021*	
C18	0.0672 (5)	0.1352 (4)	0.7717 (3)	0.0190 (9)	
H18A	0.0483	0.1155	0.7244	0.023*	
H18B	0.1585	0.1018	0.7788	0.023*	
C19	0.2302 (4)	0.2903 (4)	0.8443 (3)	0.0165 (8)	
H19A	0.2529	0.2006	0.8532	0.020*	
H19B	0.2315	0.3122	0.8941	0.020*	
C20	0.3469 (5)	0.3718 (4)	0.7746 (3)	0.0214 (10)	
H20A	0.3091	0.4512	0.7471	0.026*	
H20B	0.4233	0.3941	0.7960	0.026*	
C21	0.5325 (5)	0.2848 (4)	0.7024 (3)	0.0221 (10)	
H21	0.5900	0.3247	0.7249	0.027*	
C22	0.5967 (5)	0.2040 (5)	0.6508 (3)	0.0240 (10)	
C23	0.5150 (6)	0.1411 (5)	0.6187 (3)	0.0288 (11)	
H23	0.4183	0.1565	0.6248	0.035*	
C24	0.5744 (6)	0.0561 (5)	0.5779 (3)	0.0330 (12)	
H24	0.5180	0.0109	0.5577	0.040*	
C25	0.7162 (6)	0.0377 (5)	0.5668 (3)	0.0327 (12)	
C26	0.7993 (6)	0.1014 (6)	0.5960 (4)	0.0394 (14)	
H26	0.8970	0.0888	0.5872	0.047*	
C27	0.7394 (6)	0.1846 (5)	0.6383 (4)	0.0327 (12)	
H27	0.7963	0.2285	0.6589	0.039*	
C28	0.7789 (7)	−0.0583 (6)	0.5239 (4)	0.0425 (15)	
F4	0.8768 (6)	−0.1210 (4)	0.5533 (3)	0.0730 (14)	
F5	0.8382 (6)	−0.0061 (4)	0.4467 (3)	0.0854 (18)	
F6	0.6850 (5)	−0.1463 (6)	0.5298 (5)	0.113 (3)	

### Atomic displacement parameters (Å^2^)

**Table:**

	*U*^11^	*U*^22^	*U*^33^	*U*^12^	*U*^13^	*U*^23^
Cu1	0.0205 (3)	0.0126 (2)	0.0166 (3)	0.0093 (2)	−0.0060 (2)	−0.0075 (2)
Cl1	0.0154 (5)	0.0155 (5)	0.0217 (5)	0.0018 (4)	−0.0058 (4)	−0.0018 (4)
Cl2	0.0161 (5)	0.0132 (4)	0.0255 (6)	0.0022 (4)	−0.0049 (4)	−0.0085 (4)
N1	0.0167 (18)	0.0127 (16)	0.0169 (18)	0.0045 (14)	−0.0048 (14)	−0.0070 (14)
N2	0.0155 (17)	0.0170 (17)	0.0157 (18)	0.0062 (14)	−0.0055 (14)	−0.0076 (14)
N3	0.0206 (19)	0.0086 (16)	0.026 (2)	0.0037 (14)	−0.0063 (16)	−0.0061 (15)
N4	0.025 (2)	0.025 (2)	0.019 (2)	0.0040 (16)	−0.0080 (16)	−0.0085 (16)
C1	0.020 (2)	0.0135 (19)	0.017 (2)	0.0017 (16)	−0.0043 (17)	−0.0063 (16)
C2	0.015 (2)	0.017 (2)	0.020 (2)	0.0068 (16)	−0.0071 (17)	−0.0125 (17)
C3	0.021 (2)	0.018 (2)	0.013 (2)	0.0043 (17)	−0.0015 (16)	−0.0077 (17)
C4	0.020 (2)	0.024 (2)	0.020 (2)	0.0028 (18)	−0.0043 (18)	−0.0136 (19)
C5	0.014 (2)	0.015 (2)	0.022 (2)	0.0019 (16)	−0.0045 (17)	−0.0069 (17)
C6	0.020 (2)	0.018 (2)	0.022 (2)	0.0014 (17)	−0.0075 (18)	−0.0094 (18)
C7	0.024 (2)	0.020 (2)	0.018 (2)	0.0044 (18)	−0.0048 (18)	−0.0066 (18)
C8	0.032 (3)	0.021 (2)	0.014 (2)	−0.0002 (19)	−0.0067 (19)	−0.0018 (18)
C9	0.031 (3)	0.037 (3)	0.029 (3)	−0.001 (2)	−0.005 (2)	−0.016 (2)
C10	0.042 (3)	0.043 (3)	0.036 (3)	0.007 (3)	−0.007 (3)	−0.024 (3)
C11	0.055 (4)	0.028 (3)	0.025 (3)	0.003 (2)	−0.018 (3)	−0.011 (2)
C12	0.041 (3)	0.041 (3)	0.035 (3)	0.001 (3)	−0.020 (3)	−0.014 (3)
C13	0.033 (3)	0.034 (3)	0.026 (3)	0.005 (2)	−0.010 (2)	−0.013 (2)
C14	0.053 (4)	0.046 (4)	0.046 (4)	0.010 (3)	−0.024 (3)	−0.025 (3)
F1	0.108 (4)	0.045 (2)	0.045 (2)	−0.023 (2)	−0.017 (2)	−0.0216 (19)
F2	0.121 (4)	0.062 (3)	0.031 (2)	−0.032 (3)	−0.012 (2)	−0.0215 (19)
F3	0.114 (4)	0.085 (4)	0.142 (5)	0.048 (3)	−0.082 (4)	−0.086 (4)
Cu2	0.0214 (3)	0.0159 (3)	0.0160 (3)	−0.0060 (2)	−0.0010 (2)	−0.0079 (2)
Cl3	0.0143 (5)	0.0183 (5)	0.0204 (5)	0.0000 (4)	−0.0045 (4)	−0.0019 (4)
Cl4	0.0191 (5)	0.0137 (5)	0.0277 (6)	0.0046 (4)	−0.0064 (4)	−0.0094 (4)
N5	0.0182 (18)	0.0102 (16)	0.0143 (18)	0.0026 (13)	−0.0037 (14)	−0.0056 (14)
N6	0.0166 (18)	0.0214 (19)	0.0157 (18)	−0.0010 (14)	−0.0026 (14)	−0.0086 (15)
N7	0.0180 (18)	0.0117 (17)	0.025 (2)	0.0040 (14)	−0.0063 (15)	−0.0089 (15)
N8	0.023 (2)	0.026 (2)	0.022 (2)	0.0042 (16)	−0.0009 (16)	−0.0093 (17)
C15	0.019 (2)	0.0127 (19)	0.020 (2)	0.0063 (16)	−0.0040 (17)	−0.0097 (17)
C16	0.016 (2)	0.019 (2)	0.020 (2)	0.0005 (16)	−0.0025 (17)	−0.0126 (18)
C17	0.023 (2)	0.018 (2)	0.015 (2)	0.0040 (17)	−0.0042 (17)	−0.0103 (17)
C18	0.023 (2)	0.017 (2)	0.020 (2)	0.0055 (17)	−0.0045 (18)	−0.0114 (18)
C19	0.016 (2)	0.0143 (19)	0.020 (2)	0.0024 (16)	−0.0044 (17)	−0.0062 (17)
C20	0.020 (2)	0.018 (2)	0.025 (2)	0.0025 (17)	0.0001 (18)	−0.0085 (19)
C21	0.026 (2)	0.020 (2)	0.019 (2)	0.0013 (18)	−0.0044 (19)	−0.0053 (18)
C22	0.028 (3)	0.024 (2)	0.015 (2)	0.0053 (19)	0.0034 (18)	−0.0053 (19)
C23	0.032 (3)	0.033 (3)	0.021 (2)	0.008 (2)	−0.004 (2)	−0.011 (2)
C24	0.039 (3)	0.039 (3)	0.021 (3)	0.006 (2)	−0.005 (2)	−0.012 (2)
C25	0.036 (3)	0.030 (3)	0.026 (3)	0.005 (2)	0.005 (2)	−0.011 (2)
C26	0.025 (3)	0.041 (3)	0.049 (4)	0.008 (2)	0.003 (2)	−0.020 (3)
C27	0.026 (3)	0.033 (3)	0.039 (3)	0.001 (2)	−0.001 (2)	−0.016 (2)
C28	0.043 (3)	0.043 (3)	0.040 (3)	0.006 (3)	0.004 (3)	−0.024 (3)
F4	0.109 (4)	0.060 (3)	0.058 (3)	0.053 (3)	−0.021 (3)	−0.031 (2)
F5	0.145 (5)	0.065 (3)	0.033 (2)	0.053 (3)	0.002 (3)	−0.019 (2)
F6	0.062 (3)	0.106 (4)	0.195 (7)	−0.007 (3)	0.016 (4)	−0.124 (5)

### Geometric parameters (Å, º)

**Table:**

Cu1—N2	1.986 (3)	Cu2—N7	1.986 (4)
Cu1—N3	1.988 (4)	Cu2—N6	1.989 (4)
Cu1—N1	2.062 (4)	Cu2—N5	2.070 (4)
Cu1—Cl1	2.2701 (12)	Cu2—Cl3	2.2777 (12)
Cu1—Cl2	2.8505 (12)	Cu2—Cl4	2.9415 (12)
Cu1—Cl1^i^	3.4056 (12)	Cu2—Cl3^ii^	3.1645 (12)
N1—C1	1.480 (5)	N5—C19	1.486 (5)
N1—C3	1.493 (5)	N5—C15	1.491 (5)
N1—C5	1.496 (5)	N5—C17	1.491 (5)
N2—C2	1.491 (5)	N6—C16	1.494 (6)
N2—H2A	0.9100	N6—H6C	0.9100
N2—H2B	0.9100	N6—H6D	0.9100
N3—C4	1.478 (6)	N7—C18	1.478 (6)
N3—H3A	0.9100	N7—H7A	0.9100
N3—H3B	0.9100	N7—H7B	0.9100
N4—C7	1.260 (6)	N8—C21	1.263 (6)
N4—C6	1.466 (6)	N8—C20	1.470 (6)
C1—C2	1.518 (6)	C15—C16	1.515 (6)
C1—H1A	0.9900	C15—H15A	0.9900
C1—H1B	0.9900	C15—H15B	0.9900
C2—H2C	0.9900	C16—H16A	0.9900
C2—H2D	0.9900	C16—H16B	0.9900
C3—C4	1.516 (6)	C17—C18	1.522 (6)
C3—H3C	0.9900	C17—H17A	0.9900
C3—H3D	0.9900	C17—H17B	0.9900
C4—H4A	0.9900	C18—H18A	0.9900
C4—H4B	0.9900	C18—H18B	0.9900
C5—C6	1.529 (6)	C19—C20	1.534 (6)
C5—H5A	0.9900	C19—H19A	0.9900
C5—H5B	0.9900	C19—H19B	0.9900
C6—H6A	0.9900	C20—H20A	0.9900
C6—H6B	0.9900	C20—H20B	0.9900
C7—C8	1.482 (6)	C21—C22	1.488 (6)
C7—H7	0.9500	C21—H21	0.9500
C8—C13	1.390 (7)	C22—C27	1.390 (7)
C8—C9	1.397 (7)	C22—C23	1.394 (8)
C9—C10	1.381 (8)	C23—C24	1.386 (7)
C9—H9	0.9500	C23—H23	0.9500
C10—C11	1.377 (9)	C24—C25	1.381 (8)
C10—H10	0.9500	C24—H24	0.9500
C11—C12	1.385 (9)	C25—C26	1.373 (9)
C11—C14	1.506 (8)	C25—C28	1.519 (7)
C12—C13	1.387 (8)	C26—C27	1.389 (8)
C12—H12	0.9500	C26—H26	0.9500
C13—H13	0.9500	C27—H27	0.9500
C14—F2	1.317 (8)	C28—F4	1.306 (8)
C14—F1	1.323 (8)	C28—F5	1.312 (7)
C14—F3	1.341 (8)	C28—F6	1.318 (8)
			
N2—Cu1—N3	166.47 (15)	N7—Cu2—N6	163.80 (16)
N2—Cu1—N1	84.81 (14)	N7—Cu2—N5	85.50 (15)
N3—Cu1—N1	85.31 (14)	N6—Cu2—N5	85.18 (14)
N2—Cu1—Cl1	95.85 (11)	N7—Cu2—Cl3	95.55 (11)
N3—Cu1—Cl1	95.68 (11)	N6—Cu2—Cl3	95.56 (11)
N1—Cu1—Cl1	168.47 (11)	N5—Cu2—Cl3	171.82 (11)
N2—Cu1—Cl2	88.27 (11)	N7—Cu2—Cl4	81.07 (11)
N3—Cu1—Cl2	83.37 (11)	N6—Cu2—Cl4	86.35 (11)
N1—Cu1—Cl2	94.77 (10)	N5—Cu2—Cl4	93.74 (10)
Cl1—Cu1—Cl2	96.76 (4)	Cl3—Cu2—Cl4	94.44 (4)
N2—Cu1—Cl1^i^	115.18 (11)	N7—Cu2—Cl3^ii^	80.52 (11)
N3—Cu1—Cl1^i^	74.19 (11)	N6—Cu2—Cl3^ii^	113.16 (12)
N1—Cu1—Cl1^i^	90.95 (10)	N5—Cu2—Cl3^ii^	92.88 (10)
Cl1—Cu1—Cl1^i^	78.32 (4)	Cl3—Cu2—Cl3^ii^	79.32 (4)
Cl2—Cu1—Cl1^i^	156.30 (3)	Cl4—Cu2—Cl3^ii^	159.87 (3)
C1—N1—C3	113.3 (3)	C19—N5—C15	111.4 (3)
C1—N1—C5	112.1 (3)	C19—N5—C17	113.2 (3)
C3—N1—C5	112.9 (3)	C15—N5—C17	112.4 (3)
C1—N1—Cu1	103.3 (3)	C19—N5—Cu2	111.7 (3)
C3—N1—Cu1	104.8 (2)	C15—N5—Cu2	102.9 (3)
C5—N1—Cu1	109.8 (3)	C17—N5—Cu2	104.7 (3)
C2—N2—Cu1	111.7 (3)	C16—N6—Cu2	111.2 (3)
C2—N2—H2A	109.3	C16—N6—H6C	109.4
Cu1—N2—H2A	109.3	Cu2—N6—H6C	109.4
C2—N2—H2B	109.3	C16—N6—H6D	109.4
Cu1—N2—H2B	109.3	Cu2—N6—H6D	109.4
H2A—N2—H2B	107.9	H6C—N6—H6D	108.0
C4—N3—Cu1	110.5 (3)	C18—N7—Cu2	110.0 (3)
C4—N3—H3A	109.5	C18—N7—H7A	109.7
Cu1—N3—H3A	109.5	Cu2—N7—H7A	109.7
C4—N3—H3B	109.5	C18—N7—H7B	109.7
Cu1—N3—H3B	109.5	Cu2—N7—H7B	109.7
H3A—N3—H3B	108.1	H7A—N7—H7B	108.2
C7—N4—C6	116.6 (4)	C21—N8—C20	116.4 (4)
N1—C1—C2	109.9 (4)	N5—C15—C16	109.5 (4)
N1—C1—H1A	109.7	N5—C15—H15A	109.8
C2—C1—H1A	109.7	C16—C15—H15A	109.8
N1—C1—H1B	109.7	N5—C15—H15B	109.8
C2—C1—H1B	109.7	C16—C15—H15B	109.8
H1A—C1—H1B	108.2	H15A—C15—H15B	108.2
N2—C2—C1	109.4 (3)	N6—C16—C15	109.5 (3)
N2—C2—H2C	109.8	N6—C16—H16A	109.8
C1—C2—H2C	109.8	C15—C16—H16A	109.8
N2—C2—H2D	109.8	N6—C16—H16B	109.8
C1—C2—H2D	109.8	C15—C16—H16B	109.8
H2C—C2—H2D	108.2	H16A—C16—H16B	108.2
N1—C3—C4	108.3 (4)	N5—C17—C18	108.2 (4)
N1—C3—H3C	110.0	N5—C17—H17A	110.0
C4—C3—H3C	110.0	C18—C17—H17A	110.0
N1—C3—H3D	110.0	N5—C17—H17B	110.0
C4—C3—H3D	110.0	C18—C17—H17B	110.0
H3C—C3—H3D	108.4	H17A—C17—H17B	108.4
N3—C4—C3	108.1 (4)	N7—C18—C17	107.7 (3)
N3—C4—H4A	110.1	N7—C18—H18A	110.2
C3—C4—H4A	110.1	C17—C18—H18A	110.2
N3—C4—H4B	110.1	N7—C18—H18B	110.2
C3—C4—H4B	110.1	C17—C18—H18B	110.2
H4A—C4—H4B	108.4	H18A—C18—H18B	108.5
N1—C5—C6	116.6 (4)	N5—C19—C20	116.6 (4)
N1—C5—H5A	108.1	N5—C19—H19A	108.2
C6—C5—H5A	108.1	C20—C19—H19A	108.2
N1—C5—H5B	108.1	N5—C19—H19B	108.2
C6—C5—H5B	108.1	C20—C19—H19B	108.2
H5A—C5—H5B	107.3	H19A—C19—H19B	107.3
N4—C6—C5	110.1 (4)	N8—C20—C19	109.5 (4)
N4—C6—H6A	109.6	N8—C20—H20A	109.8
C5—C6—H6A	109.6	C19—C20—H20A	109.8
N4—C6—H6B	109.6	N8—C20—H20B	109.8
C5—C6—H6B	109.6	C19—C20—H20B	109.8
H6A—C6—H6B	108.1	H20A—C20—H20B	108.2
N4—C7—C8	121.4 (4)	N8—C21—C22	120.6 (5)
N4—C7—H7	119.3	N8—C21—H21	119.7
C8—C7—H7	119.3	C22—C21—H21	119.7
C13—C8—C9	118.8 (5)	C27—C22—C23	119.1 (5)
C13—C8—C7	119.6 (4)	C27—C22—C21	119.5 (5)
C9—C8—C7	121.5 (5)	C23—C22—C21	121.3 (4)
C10—C9—C8	120.3 (5)	C24—C23—C22	120.2 (5)
C10—C9—H9	119.9	C24—C23—H23	119.9
C8—C9—H9	119.9	C22—C23—H23	119.9
C11—C10—C9	120.1 (5)	C25—C24—C23	119.5 (5)
C11—C10—H10	120.0	C25—C24—H24	120.2
C9—C10—H10	120.0	C23—C24—H24	120.2
C10—C11—C12	120.7 (5)	C26—C25—C24	121.1 (5)
C10—C11—C14	119.3 (6)	C26—C25—C28	120.0 (5)
C12—C11—C14	120.0 (6)	C24—C25—C28	118.8 (5)
C11—C12—C13	119.0 (5)	C25—C26—C27	119.4 (5)
C11—C12—H12	120.5	C25—C26—H26	120.3
C13—C12—H12	120.5	C27—C26—H26	120.3
C12—C13—C8	121.0 (5)	C26—C27—C22	120.5 (5)
C12—C13—H13	119.5	C26—C27—H27	119.7
C8—C13—H13	119.5	C22—C27—H27	119.7
F2—C14—F1	105.8 (5)	F4—C28—F5	105.2 (5)
F2—C14—F3	107.4 (6)	F4—C28—F6	104.6 (6)
F1—C14—F3	103.8 (6)	F5—C28—F6	107.6 (6)
F2—C14—C11	113.2 (5)	F4—C28—C25	113.6 (6)
F1—C14—C11	113.8 (6)	F5—C28—C25	112.7 (5)
F3—C14—C11	112.1 (5)	F6—C28—C25	112.5 (5)
			
C3—N1—C1—C2	161.1 (3)	C19—N5—C15—C16	−71.4 (4)
C5—N1—C1—C2	−69.8 (4)	C17—N5—C15—C16	160.5 (4)
Cu1—N1—C1—C2	48.3 (4)	Cu2—N5—C15—C16	48.4 (4)
Cu1—N2—C2—C1	17.1 (4)	Cu2—N6—C16—C15	19.9 (4)
N1—C1—C2—N2	−44.7 (5)	N5—C15—C16—N6	−46.7 (5)
C1—N1—C3—C4	−156.5 (4)	C19—N5—C17—C18	77.8 (4)
C5—N1—C3—C4	74.8 (4)	C15—N5—C17—C18	−155.0 (4)
Cu1—N1—C3—C4	−44.7 (4)	Cu2—N5—C17—C18	−44.0 (4)
Cu1—N3—C4—C3	−33.0 (4)	Cu2—N7—C18—C17	−35.9 (4)
N1—C3—C4—N3	52.3 (5)	N5—C17—C18—N7	54.0 (5)
C1—N1—C5—C6	−70.5 (5)	C15—N5—C19—C20	−72.8 (5)
C3—N1—C5—C6	58.9 (5)	C17—N5—C19—C20	55.0 (5)
Cu1—N1—C5—C6	175.4 (3)	Cu2—N5—C19—C20	172.7 (3)
C7—N4—C6—C5	−120.4 (4)	C21—N8—C20—C19	−122.6 (5)
N1—C5—C6—N4	−84.7 (5)	N5—C19—C20—N8	−89.7 (4)
C6—N4—C7—C8	173.7 (4)	C20—N8—C21—C22	173.9 (4)
N4—C7—C8—C13	−172.1 (5)	N8—C21—C22—C27	−178.1 (5)
N4—C7—C8—C9	3.0 (7)	N8—C21—C22—C23	−1.9 (7)
C13—C8—C9—C10	2.4 (8)	C27—C22—C23—C24	2.8 (8)
C7—C8—C9—C10	−172.7 (5)	C21—C22—C23—C24	−173.4 (5)
C8—C9—C10—C11	0.7 (9)	C22—C23—C24—C25	−2.3 (8)
C9—C10—C11—C12	−3.1 (9)	C23—C24—C25—C26	0.4 (9)
C9—C10—C11—C14	175.8 (6)	C23—C24—C25—C28	178.4 (5)
C10—C11—C12—C13	2.3 (9)	C24—C25—C26—C27	0.9 (9)
C14—C11—C12—C13	−176.6 (5)	C28—C25—C26—C27	−177.0 (6)
C11—C12—C13—C8	0.9 (8)	C25—C26—C27—C22	−0.4 (9)
C9—C8—C13—C12	−3.2 (8)	C23—C22—C27—C26	−1.5 (8)
C7—C8—C13—C12	172.0 (5)	C21—C22—C27—C26	174.8 (5)
C10—C11—C14—F2	84.1 (7)	C26—C25—C28—F4	34.7 (8)
C12—C11—C14—F2	−97.1 (7)	C24—C25—C28—F4	−143.3 (6)
C10—C11—C14—F1	−155.1 (6)	C26—C25—C28—F5	−84.8 (8)
C12—C11—C14—F1	23.8 (8)	C24—C25—C28—F5	97.2 (7)
C10—C11—C14—F3	−37.6 (9)	C26—C25—C28—F6	153.2 (7)
C12—C11—C14—F3	141.2 (6)	C24—C25—C28—F6	−24.8 (9)

Symmetry codes: (i) −*x*+1, −*y*+1, −*z*; (ii) −*x*, −*y*, −*z*+2.

### Hydrogen-bond geometry (Å, º)

**Table:**

*D*—H···*A*	*D*—H	H···*A*	*D*···*A*	*D*—H···*A*
N2—H2*A*···Cl3^iii^	0.91	2.84	3.591 (4)	141
N2—H2*B*···Cl4^iv^	0.91	2.46	3.365 (4)	171
N3—H3*A*···Cl1^i^	0.91	2.95	3.444 (4)	115
N3—H3*A*···Cl4^v^	0.91	2.60	3.334 (4)	139
N3—H3*B*···Cl2	0.91	2.83	3.281 (4)	112
N6—H6*C*···Cl1^v^	0.91	2.96	3.681 (4)	138
N6—H6*D*···Cl2^vi^	0.91	2.45	3.342 (4)	167
N7—H7*A*···Cl2^iii^	0.91	2.57	3.348 (4)	143
N7—H7*B*···Cl4	0.91	2.79	3.284 (4)	115

Symmetry codes: (i) −*x*+1, −*y*+1, −*z*; (iii) −*x*, −*y*, −*z*+1; (iv) *x*+1, *y*, *z*−1; (v) −*x*, −*y*+1, −*z*+1; (vi) *x*, *y*, *z*+1.
