# Supplementary material for: Crystal structure of [bis­(2-amino­ethyl-κN)(2-{[4-(tri­fluoro­meth­yl)benzyl­idene]amino}­eth­yl)amine-κN]di­chlorido­copper(II)
Source: Acta Crystallogr E Crystallogr Commun. 2016 Jan 1;72(Pt 1):83–6. doi: 10.1107/S2056989015024147 (PMC4704757; doi:10.1107/S2056989015024147)
Supplement: Supplementary file 3 [file e-72-00083-Isup3.pdf]

1. The asymmetric unit as chosen does not match with the way in which the structure is described. This is most notable in the position of Cl4, which ought to be located in the model close to Cu2. The refined coordinates of (0.300919, 0.730937, 0.132890) would need to be transformed through an to (-x,1-y,1-z) in order to be consistent with the discussion of geometry in the Abstract. Crystallographically speaking, it doesn't matter, but aesthetically speaking it would make much more sense.

Author reply: Thank you for pointing this out. Cl4 is now "close" to Cu2. We elected to leave it in the observed position because it is comparable with the Cu1-Cl2 long contact. The longest Cu…Cl interactions are across the independent inversion centers and are Cu1…Cl1' and Cu2…Cl3". We have revised the abstract to better reflect this discussion.

2. The very long Cu...Cl distances that would constitute the sixth position for the very elongated octahedral are still not addressed in the Abstract. If one opens the structure in Mercury and displays the 'short contacts', these geometric features become quite clear, but the Abstract does not mention them.

Author reply: Our apologies for this oversight, we have revised the abstract to better reflect the unusual coordination geometry about the Cu centers.

3. Standard uncertainties on distances in addition to the primary bonding interactions can be obtained as part of a SHELXL refinement using the RTAB command. I don't use OLEX2, so I have no idea how one might do it in that program, but since it appears to use SHELXL as its refinement engine, I assume you could simply edit the text files to add appropriate RTAB instructions.

Author reply: We elected to use BIND, coupled with appropriate EQIV commands to generate the contacts to the elongated Cu1/2---Cl2/4 and Cu1/2….Cl1'/3" with s.u.'s

4. The way the asymmetric unit is now arranged, you would also need an EQIV statement to define the transformation to shift Cl4 (for example). You would also have to use EQIV statements for the extra 'very long distances' between Cu1 and a copy of Cl1 and Cu2 and a copy of Cl3, as per the faint dotted line in the new Fig. 3.

Author reply: Cl4 seemed to be "proximal" to Cu2. We elected to leave it in the current location (see RES and coordinates). Please see above for generated bonds.

5. In the Abstract and elsewhere, quoted distances that are derived from the refinement need to have their SU values quoted. To obtain SUs for the 'elongations' mentioned, standard propagation of errors will work, but it is probably better to give the actual Cu...Cl distances. The 'elongations' mentioned in the text seem to be relative to the normal Cu-Cl bonds, but each uses a different starting point Cu-Cl distance (i.e., either the normal Cu1-Cl1 or Cu2-Cl3 distances), and so the discussion seems a bit muddled.

Author response: Our apologies for this, we have clarified the statements and included s.u.'s on all distances and angles stated within the body text. Hopefully the text re-organization now has a better flow that can be easily followed by readers.

6. The disorder occupancies would also require SUs, as would the distances and angles mentioned in the 'Supramolecular features' section.

Author response: The model was re-examined and we found that the model was satisfactory with slightly elongated atomic displacement ellipsoids for the fluorine atoms; there was no real, useful or new information to be obtained by modeling the disorder over two sites. The significant residual electron density is located near the Cu centers in any case, and only a 1.1 e- peak is near the CF3 groups. The new model, discussion and figures have been updated to account for this.

7. The 'very elongated' Cu...Cl distances mentioned in the first review still needs further clarification because I'm not sure that the present text describes them in sufficient detail. Whatever their origin (Jahn-Teller elongation, electrostatic, van der Waals etc.), these interactions appear to be real and have some structure-directing properties. It looks to me as though these leads to dimers of each of the unique molecules, and then it is these dimers that associate via stacking of the aromatic rings to form extended chains. I think you'd need to state the distances (with SUs) and give the symmetry operations that relate the parts. This would have to be added to the Abstract and to the Structural commentary section.

Author response: Thank you for pointing this out, we have subsequently revised the commentary to describe the interactions a bit more carefully. We agree that there are definitely structure-directing interactions; both as dimers and further through pi…-pi interactions. We hope that the clarification applied to the text is appropriate.

9. Supramolecular features: This ought to be consistent with the Structural commentary, which seems to imply that the elongated Cu...Cl are more electrostatic in nature rather than Jahn-Teller distortions.

Author response: As above; we hope that the new discussion has been revised to be more appropriate and better describe the rather unusual interactions within these molecules.

10. Supramolecular features: SU needed on the centroid-centroid distance and interplanar ring dihedral.

Author response: corrected as requested.

11. Supramolecular features: The propagation of these chains is not along [100]. It looks more like [021] to me, but please check this carefully.

Author response: Our apologies for this error, we agree and the text has been modified to correct for this.

12. Refinement: For the DFIX and DANG restraints, please also give the assigned uncertainty.

Author response: Because we have removed the disorder these restraints are no longer applied.

13. There is an over generous table of 'hydrogen bonds' provided. Many of the 'hydrogen bonds' seem to be merely a consequence of the proximity of the atoms in question. Indeed, none of the interactions listed in Table 2 are

identified as hydrogen bonds using the default settings in Mercury, which is usually quite good at such things. Please bear in mind that SHELXL-2014 is very generous and over inclusive when it flags potential hydrogen bonds, and in this case, many of them seem to be false positives. These interactions should be analyzed in detail and the spurious ones need to be removed from the discussion.

Author response: We have pruned down the H-bond interactions to a more reasonable level. The interactions we have opted for solely include N-H…Cl contacts as viable H-bond interactions. Discussion has also been added to the Supramolecular Features section highlighting these.

14. Refinement: Replace 'thermal values' with either 'thermal parameters' or 'displacement parameters'. The latter is probably more current usage.

Author Response: This has been done, thank you for the suggestion.

15. Fig. 1 caption: Since the atoms are labelled, the description of colour coding is superfluous.

Author Response: This has been corrected.
